# Differences in Admission Rates of Children with Pneumonia Between Pediatric and Community Emergency Departments

**DOI:** 10.5811/westjem.47221

**Published:** 2025-11-26

**Authors:** Grace VanGorder, Samuel Lee, Zachary Jensen, Susan Boehmer, Robert P. Olympia

**Affiliations:** Penn State University College of Medicine, Department of Emergency Medicine, Hershey, Pennsylvania

## Abstract

**Introduction:**

Pneumonia is the most common cause of pediatric death worldwide. We sought to determine whether the rate of hospital admission of pediatric patients diagnosed with pneumonia at a dedicated pediatric-emergency department (PED) is different than the rate at a community emergency department (CED). This comparison may provide insight into decision-making and factors associated with admission.

**Methods:**

In this retrospective cohort study we reviewed patient records from January 1, 2017–December 31, 2019 for pediatric patients diagnosed with pneumonia. We excluded patients who were not prescribed antibiotics, those who did not receive a chest radiograph or had no radiologic signs of pneumonia. In addition, we excluded patients with comorbid conditions such as tracheostomy, supplemental oxygen requirement at baseline, chronic lung disease other than asthma or reactive airway disease, any cancer diagnosis, cystic fibrosis, or congenital heart disease. The primary outcome was the proportion of pneumonia diagnoses that resulted in admission from the PED vs CED. We used logistic regression analyses to evaluate which clinical factors were associated with hospital admission. Significance levels were determined by chi-square test or the Fisher exact test and Cochran-Mantel-Haenszel statistic.

**Results:**

We identified 400 pediatric patients with pneumonia, 182 from the PED and 218 from the CED. There was a significant difference in admission rates between the two hospitals: 53 of 182 patients in the PED were admitted (29.1%) vs 27 of 218 patients in the CED (12.4%, *P* < .001). Patients in the PED were, therefore, 2.35 times more likely to be admitted than those at the CED (odds ratio 5.1, 95% CI, 2.5–10.4). Patients presenting to the PED were more likely to arrive via ambulance (10.7% vs 3.1%, *P* = .04) and to be hypoxic upon arrival (13.2% vs 3.2%, *P* < .001). The median age of patients in the PED was significantly higher than the CED (6.0 years vs 2.0 years, *P* < .001). A significantly greater proportion of patients in the CED identified as Hispanic or Latino (68.6% vs 20.3%, *P* < .001). Patients in the CED were more likely to be insured (11.0% vs 19.9%, P = .01). There was no significant difference in immunization status between the two groups.

**Conclusion:**

Patients presenting to a dedicated pediatric ED had a higher admission rate than did those at a community ED. Patients in the PED were more likely to arrive by ambulance and less likely to have active health insurance coverage. Patients at the PED were more likely to be hypoxic than patients at the CED. These findings highlight important practice differences between PEDs and CEDs that may inform strategies to improve patient outcomes, reduce costs, and promote more effective, evidence-based care. Future studies should further investigate the drivers of these variations and evaluate targeted interventions to optimize care across settings.

## INTRODUCTION

Each year, over 100,000 children in the United States are hospitalized due to pneumonia.^1^,^2^ This accounts for 1–4% of all emergency department (ED) visits.^3^,^4^ The Pediatric Infectious Disease Society recommends that patients with moderate to severe community-acquired pneumonia (evidenced by tachypnea, clinical signs of dyspnea, altered mental statu, s or hypoxemia with pulse oximetry measurement < 90%) be admitted for inpatient treatment. Additional recommendations include that any patient < 3 months of age with suspected bacterial pneumonia, any child with suspected community-associated methicillin-resistant pneumonia, or children for whom there is concern about careful observation or therapy adherence at home be admitted.^5^

Various factors influence the risk of infection. Infants and children < 5 years of age are at heightened risk due to their developing immune systems and smaller airways, which are more susceptible to obstruction. Children with underlying health conditions such as chronic respiratory diseases, immunocompromised states, and cardiovascular or neuromuscular disorders are at increased risk as well. Exposure to second-hand smoke, air pollution, and crowded living conditions, such as in daycare settings, significantly raises the likelihood of pneumonia infection and subsequent hospital admission. Malnutrition and vitamin deficiencies can weaken immune function. Children who are not fully vaccinated against pathogens like *Streptococcus pneumoniae*, *Haemophilus influenzae*, and the influenza virus are particularly vulnerable.^6^ The interaction between host factors and the pathogen, including genetic predispositions and the type of infecting organism, further influences the severity of pneumonia.

Socioeconomic factors also contribute, as children from lower income backgrounds may face barriers to healthcare and encounter poorer living conditions. Racial and ethnic disparities exist in pneumonia treatment and outcomes. Non-Hispanic Black children have been reported to experience higher rates of pneumonia-related complications compared to non-Hispanic White children.^7^ Understanding these multifaceted risk factors impacting the disease process and likelihood of admission is crucial in guiding preventive strategies, such as vaccination, reducing environmental exposures, and improving healthcare access to mitigate the burden of pediatric pneumonia.

No previous studies have been performed comparing admission rates specifically for pediatric pneumonia between community EDs (CED) and pediatric EDs (PED), although previous researchers have compared pediatric cohorts in tertiary-care centers vs CEDs for conditions other than pneumonia (eg, migraines).^8^ A CED primarily focuses on providing immediate medical care for a wide range of medical concerns for adults and children within a specific geographic area, while the PED specializes in treating children with acute illnesses and injuries, often within a larger academic medical center that emphasizes research and teaching. Bourgeois et al identified significant variation—up to threefold—in hospital-level admission rates for pediatric pneumonia at pediatric tertiary-care hospitals across the US, highlighting the need for greater standardization of admissions decisions.^9^ If such wide differences were found to exist between tertiary-care centers, it is likely that similar gaps exist between community and tertiary-care centers.

**Population Health Research Capsule**

What do we already know about this issue?

*There is significant variation in hospital admission rates for pneumonia patients at pediatric tertiary-care hospitals.*

What was the research question?

*Is the rate of hospital admission of pediatric patients with pneumonia different at a pediatric emergency department (PED) vs a community emergency department (CED)?*

What was the major finding of the study?

*Among pediatric patients with pneumonia, admission rates were significantly higher in PEDs compared to CEDs (29.1% vs 12.4%; CI, 2.5–10.4).*

How does this improve population health?

*Understanding these dynamics can potentially reduce unnecessary hospitalizations at both PEDs and CEDs while ensuring that more severe cases receive the necessary care.*

## AIMS AND OBJECTIVES

In this study we sought to determine whether the rate of hospital admission of pediatric patients diagnosed with pneumonia at a dedicated PED was different than the rate at a CED. Emergency physicians at PEDs may be more comfortable with or experienced in managing pediatric pneumonia, due to increased volume and higher rates of pediatric fellowship-trained physicians relative to their CED counterparts. As a result, these pediatric emergency physicians may have greater confidence in discharging patients home rather than admitting. However, because of their status as a pediatric center, PEDs may receive proportionally sicker patients, inflating the admission rate. We compared data from a PED and a CED to analyze differences in admission, excluding patients with pre-existing immunodeficiencies or lung diseases to control for selection bias of patients’ families toward a tertiary-care center. We hypothesized that the proportion of pediatric patients with pneumonia admitted to the hospital would be significantly lower at the PED than at the CED.

## METHODS

The PED in this study is a Level I pediatric trauma center with a children’s hospital offering specialized pediatric emergency care. The PED, which serves both rural and urban communities, is 15 minutes from a major city. This center is staffed by board-certified pediatric emergency physicians. In 2023, the PED managed 24,236 patient visits, while the adult ED in the same institution handled 55,319 visits. The CED is a 204-bed acute care hospital serving a suburban community. It is staffed by board-certified emergency physicians. The center, which does not have a dedicated pediatric ED or trauma center designation, sees approximately 36,170 adult and pediatric patients annually.

In conducting this study, we referenced the framework for retrospective chart review in emergency medicine research outlined by Worster and Bledsoe. We included the following elements: abstractor training; case selection criteria; variable definition; abstraction forms; medical record identification; and institutional review board approval.^10^ Abstractors were not blinded to the study hypothesis, as the main data abstractor was also responsible for the study design. Additionally, we did not formally assess inter-rater reliability, as most of the data collection was performed by a single individual. In this retrospective cohort study we used patient charts over the period of January 1, 2017–December 31, 2019. This time frame was selected as it was the most current data available at the time of project initiation and was prior to the start of the COVID-19 pandemic, which would have introduced a variety of confounding factors for analysis.

To be included in the study, a patient must have been < 18 years of age at the time of the ED visit and diagnosed with pneumonia (*International Classification of Diseases, 10^th^ Modification* codes J12–J18) by chest radiograph (CXR). The CXRs were interpreted by radiologists to be suggestive of pneumonia if they showed consolidation, patchy infiltrates, atelectasis, or were otherwise determined by the radiologist to be suggestive of pneumonia. We reviewed the finalized impression summary and extracted key words, which were a part of the analysis. We excluded patients who were not prescribed antibiotics, did not receive a CXR or if they received a CXR without radiographic signs of pneumonia. We also excluded any patient with pre-existing conditions impacting lung function or immune response to control for bias for patients who were likely to become more ill from a pneumonia infection to present to the higher acuity center and, subsequently, increase their admission rates. These conditions included chronic lung disease, heart failure, previous heart or lung surgery, tracheostomy placement, or use of supplemental oxygen at baseline. Asthma and reactive airway disease were not controlled for in this study because diagnosis of asthma has not been strongly correlated with pneumonia outcomes. Although there is a correlation between early pneumonia infection and subsequent development of asthma by the age of four,^11^ patients with pre-existing asthma tend to have clinical outcomes similar to the general population.^12^

All patient encounters meeting inclusion criteria during the period of interest were identified by our institutions’ enterprise information management, which then provided the researchers with medical record numbers and basic encounter information. Each encounter was considered separately. For example, if a patient was diagnosed with pneumonia, discharged, and returned days later with clinical worsening, they would be included as two separate encounters, as inititally the child did not meet the institution’s criteria for admission but in the second encounters did meet the criteria. The primary outcome was the proportion of pneumonia diagnoses that results in admission from PEDs vs CEDs. Secondary outcomes included demographics, clinical characteristics, and comorbid conditions. Data abstractors were trained on the inclusion and exclusion criteria and data collection protocols.

Study data were collected and managed using REDCap electronic data capture tools hosted at Penn State University. REDCap (Research Electronic Data Capture) is a secure, web-based software platform designed to support data capture for research studies.^13^,^14^ This study was approved by our institutional review board (STUDY 00018516).

We conducted a power analysis to determine the appropriate sample size, resulting in a selection of 400 participants to detect a 15% difference with adequate statistical power. Variables evaluated include age, sex, clinical severity (determined by hypoxemia, dyspnea, tachycardia, altered mental status, temperature, dehydration, decreased perfusion, hypotension, duration of symptoms), medical comorbidities, and CXR factors (unilateral, bilateral, focal infiltrate, atelectasis, presence of pleural effusion, etc). Descriptive statistics (means, medians, standard deviations) were generated for continuous variables. We used logistic regression analyses to evaluate criteria associated with hospital admission. Differences between cohorts were characterized using contingency table analysis; significance levels were determined by chi-square statistic, the Fisher exact test and Cochran-Mantel-Haenszel statistic. For this study, significant differences were determined at *P* < .05. We used appropriate non-parametric procedures in the cases of small sample sizes, and we used SAS v9.4 (SAS Institute, Inc, Cary, NC) for data analysis.

## RESULTS

**Figure 1:**
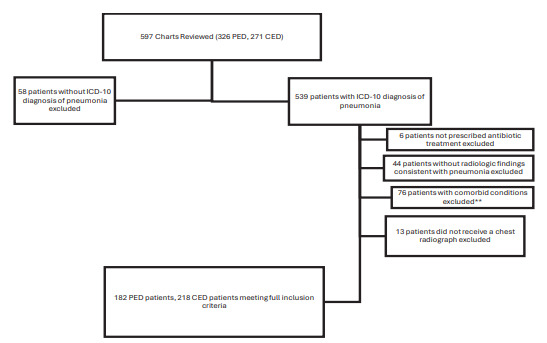
Flowchart of inclusion and exclusion criteria used to determined how pediatric patients with pneumonia at a pediatric emergency department and community emergency department were selected for inclusion in the study. **Previous cancer diagnosis, chronic lung disease (not including asthma or reactive airway disease), heart failure, previous heart or lung surgery, tracheostomy placement, or use of supplemental oxygen at baseline. *CED*, community emergency department; *PED*, pediatric emergency department; *ICD-10*, *International Classification of Diseases, 10^th^ Mod*.

To address our objective, we identified patient encounters that met our inclusion criteria and compared rates of admission, patient characteristics, and factors impacting illness severity between sites. Analysis was performed on 400 pediatric patients who met inclusion criteria and were logged into REDCap (Figure 1). We reviewed 182 PED encounters and 218 CED encounters between January 1, 2017–December 31, 2019. Of the 182 patients presenting to the PED, 129 (70.9%) were discharged and 53 (29.1%) required inpatient hospital admission. Of the 218 presenting to the CED, 191 (87.6%) were discharged and 27 (12.4%) admitted (Table 1). These data indicate a notably higher admission rate at the PED compared to the CED. Patient demographics, insurance type, and clinical risk factors for pneumonia, such as history of asthma, prematurity, and secondhand smoke exposure, are presented in Table 2. Overall, the cohorts were comparable in terms of age and sex distribution. We observed differences in insurance status and presence of certain risk factors, such as previous asthma diagnosis.

Clinical features on presentation, including initial vital signs, oxygen saturation, and other indicators of clinical severity, are summarized in Table 3. Patients presenting to the PED were more likely to exhibit abnormal vital signs, including tachypnea and hypoxia than those at the CED. To evaluate for differences in admission rates between the. PED and CED, we performed a chi-square test of independence, yielding a significant result (χ^2^(1) = 22.2, *P* < .001, ϕ = −0.37). This suggests a statistically significant result with moderate-to-large effect size. A logistic regression analysis comparing admission rates between systems likewise yielded a significant result (χ^2^(1) = 20.6, *P* < .001). Patients at the PED had a significantly higher likelihood of admission compared to those at the CED. The admission rate at the PED was 29.1%, whereas at the CED it was 12.4%. The odds of admission at the PED were 5.1 times higher than at the CED (odds ratio 5.1, 95% CI, 2.5–10.4), indicating a substantial difference in admission practices between the two hospitals.

## DISCUSSION

In this study we aimed to answer whether there was a significant difference in pediatric pneumonia admission rates between CEDs and PEDs. The results demonstrate that the odds of admission to the PED for pneumonia were 2.35 times greater than to the CED. This denotes a significantly higher admission rate for pediatric patients with pneumonia at the PED compared to the CED, aligning with our objective to explore how the type of medical institution may influence clinical decision-making. Additionally, patients presenting to the PED were more likely to be admitted to the ED observation unit than those at the CED. The observation unit allows for extended monitoring and additional workup for patients who do not meet criteria for hospital admission but may be inappropriate for discharge. The rate of admission at the. PED was higher likely due to patients being sicker upon arrival. There were several key factors contributing to this, most notably the following statistically significant differences between the two sites including higher rates of hypoxia and more patients requiring fluid resuscitation for hemodynamic instability among PED patients. Patients presenting to the PED were also more likely to arrive via ambulance (10.7% vs 3.1%).

Dean and Florin noted in their systematic review that tachycardia may be associated with greater clinical severity of pneumonia.^15^ There was no significant difference on the auscultation of wheezing on physician examination between the two sites. Wheezing is a component of the Novel Pneumonia Risk Score, a scoring tool developed to identify low-risk patients for whom radiography and antibiotics can be avoided. Its presence decreases the likelihood of a pneumonia that requires imaging and antibiotics.^16^ We were unable to demonstrate the importance of this factor with our study population. We did, however, note that physicians auscultated crackles or rales more frequently in the PED cohort, which could have been associated with hospital admission. Nevertheless, auscultation is a subjective measure that can vary based on the examiner.

The findings of this study reveal a notable difference in admission rates between the PED and the CED, with the PED admitting 29.1% of pediatric pneumonia patients compared to 12.4% admitted at the CED. This negates the hypothesis that emergency physicians in the PED, who have more pediatric-specific training and experience, may be more comfortable managing pneumonia cases on an outpatient basis. Our study contradicts previous research studies, which identified several factors as higher risk indicators for pneumonia and are often associated with higher rates of admission, including being **uninsured**, **Black/Non-Hispanic**, **age**, and **vaccination status**. Despite these well-known risk factors, we did not observe a higher admission rate at the PED among patients with these characteristics. This discrepancy raises questions about the impact of **socioeconomic factors** on admission decisions and suggests that other variables not controlled for—such as physician experience, institutional practices, or referral patterns—may play a more significant role in the admission process.

The median age of patients at the PED was significantly higher (6.0 vs 2.0 years, *P* < .001), suggesting that younger patients, who may be more vulnerable to severe pneumonia, were more likely to present to the CED. Patients presenting to the CED were more often infants and toddlers. Additionally, a significantly greater proportion of patients at the CED identified as Hispanic or Latino (68.6% vs 20.3%, *P* < .001). They were less likely to be uninsured (11.0% vs 19.9%, *P* = 0.01). These findings raise questions about potential socioeconomic factors influencing admission decisions. Clinical presentation also varied, with dyspnea reported more frequently at the PED (19.1% vs 6.6%, *P* < .001), which may have contributed to higher admission rates. These findings suggest that both physician experience and patient population characteristics play a role in admission decisions, warranting further investigation into the impact of physician training, institutional resources, and social determinants of health on pediatric pneumonia management.

## LIMITATIONS

Despite its valuable insights, this study has several limitations. First, the retrospective nature of the analysis introduces potential biases, including incomplete documentation and variability in clinical decision-making that cannot be fully accounted for. Additionally, the study does not assess clinician-level differences, such as variations in training, experience, or institutional admission protocols, which may have influenced admission rates independently of the hospital setting. The study was also limited in that it only compared one CED site to one PED site. Differences in institutional protocols, patient population, and clinician experience may limit the generalizability of our findings to other settings. Another limitation is the demographic and socioeconomic differences between patient populations at the two hospitals. The significantly higher proportion of Hispanic and uninsured patients at the CED suggests that healthcare access and social determinants may play a role in admission decisions, yet these factors were not directly analyzed. Furthermore, illness severity was not consistently measured across all patients, making it difficult to determine whether differences in admission rates reflected physician confidence or true differences in disease burden.

Data abstraction yielded nearly 400 patient encounters meeting inclusion criteria, with about 200 encounters per institution. As per the sample-size calculations, this sample size provided enough power to identify a difference of more than 15% in admission rates between the two institutions. To assess secondary outcomes more fully with more statistical power, larger sample sizes will be required.

Subsequent research should evaluate the treatment course and outcomes for admitted patients. Comparing length of stay, interventions required, and return to the ED following discharge would provide insight into the admission practices at each institution to evaluate which institution is most appropriately triaging pediatric patients with pneumonia. Future research should also aim to address these limitations by incorporating a prospective design with standardized severity scoring to better control for clinical presentation. A multicenter study including additional PED and CED settings would also help generalize findings and identify broader trends in pediatric pneumonia management. Additionally, qualitative research on physician decision-making, as well as an analysis of post-discharge outcomes, could provide further insights into the safety and effectiveness of outpatient management. Exploring the impact of social determinants, such as language barriers and healthcare access, on admission decisions could also inform strategies for equitable pediatric care.

## CONCLUSION

This study demonstrates that the admission rate for pediatric pneumonia patients was higher at a pediatric than at a community emergency department, contrary to the initial hypothesis. This study provides important insights into pediatric pneumonia management at two distinct EDs. Although we found a higher admission rate at the PED, the results were influenced by the sicker patient population, as evidenced by factors like hypoxia, the need for intravenous fluids, and arrival by ambulance. Further analysis, controlling for these severity factors, is necessary to determine whether the observed differences are due to institutional practices or differences in patient severity.

The results of this study could provide insight in the variability in clinical practice between PEDs and CEDs and have implications for differences in healthcare costs, antibiotic stewardship, and hospital-acquired infection rates. This insight could impact future patient care in these settings and help lead to better and more cost-effective care. Understanding these dynamics could help improve decision-making and clinical practices in both CED and PED settings, potentially reducing unnecessary hospitalizations while ensuring that more severe cases receive the necessary care.

## Supplementary Information



## Figures and Tables

**Table 1:** Rate of admission to inpatient hospital status of children with pneumonia to a pediatric vs community emergency department.

	Admission Status				Rate of Hospitalization	*P*-value*
		Admit				
	Discharge	Inpatient	ICU	Total		
PED	129/182 (70.8%)	46/182 (25.2%)	7 (3.8%)	182	29.1%	
CED	191/218 (87.6%)	27/218 (12.4%)	-	218	12.4%	
Total	320	73	7	400	20.0%	< .001

Emergency department disposition for pediatric patients presenting with pneumonia to a pediatric emergency department and a community emergency department.

* Corresponds to *t*-test for continuous variables and chi-square test for categorical variables.

*CED*, community emergency department; *ICU*, intensive care unit; *PED*, pediatric emergency department.

**Table 2:** Demographics, insurance status, and pneumonia risk factors for children presenting to a pediatric vs community emergency department.

N	PED	CED	Overall	*P*-value*
	182	218	400	
Biological sex				.87
Male	105 (57.7%)	124 (56.8%)	229	
Female	77 (42.3%)	94 (43.1%)	171	
Age, years				< .001
Median [IQR]	6.0 [3.0–9.0]	2.0 [1.0–5.0]	4.0 [1.0–7.0]	
Ethnicity				< .001
Hispanic, Latino, and/or Spanish origin	37 (20.3%)	149 (68.6%)	186	
Not Hispanic, Latino, and/or Spanish origin	141 (77.8%)	64 (29.5%)	205	
Declined to answer or unknown	4 (2.2%)	4 (1.8%)	8	
Race				
White	111 (61.0%)	49 (22.5%)	160	< .001
Black	8 (4.4%)	15 (6.9%)	23	.28
American Indian and/or Alaska Native	1 (0.6%)	0 (0.0%)	1	.27
Asian	8 (4.4%)	0 (0.0%)	8	-
Native Hawaiian and/or Pacific Islander	0 (0.0%)	0 (0.0%)		-
Other race	29 (15.9%)	141 (64.7%)	170	< .001
Declined to answer or unknown	7 (3.9%)	6 (2.8%)	13	.54
Insurance status				.01
Uninsured	36 (19.9%)	24 (11.0%)	60	
Insured	145 (80.1%)	194 (89.0%)	339	
Premature birth (< 36 weeks gestation)				**
Yes	14 (9.5%)	13 (6.3%)	27	
No	20 (13.5%)	58 (28.2%)	78	
Not documented	114 (77.0%)	135 (65.5%)	249	
Smoke exposure				
No smoke exposure at home	37 (20.3%)	30 (13.8%)	67	.08
Patient smokes	2 (1.1%)	0 (0.0%)	2	.12
Other smoker in the home	10 (5.5%)	7 (3.2%)	17	.26
Not documented	123 (67.6%)	175 (80.3%)	298	< .01
Vaccination status				.53
Up to date	98 (54.1%)	113 (51.8%)	211	
Not up to date	7 (3.9%)	5 (2.3%)	12	
Not documented	76 (42.0 %)	100 (45.9%)	176	
History of Asthma				.02
Yes	45 (24.7%)	34 (15.6%)	79	
No	137 (75.3%)	184 (84.4%)	321	

Demographic comparisons for pediatric patients presenting to the emergency department with diagnosis of pneumonia at a community emergency department and a pediatric emergency department.

* Corresponds to *t*-test for continuous variables and chi-square test for categorical variables.

- Not applicable or sample too small.

** Sample sizes are not representative of incidence/prevalence within a single institution due to incomplete data

*CED*, community emergency department; *IQR*, interquartile range; *PED*, pediatric emergency department.

**Table 3:** Clinical features of children with pneumonia presenting to a pediatric vs community emergency department.

N	PED	CED	Overall	*P*-value*
	182	218	400	
Arrival by ambulance				< .01
Yes	17 (10.7%)	6 (3.1%)	23	
No	142 (89.3%)	189 (96.9%)	159	
Chief complaint				< .001
Fever	63 (37.5%)	90 (42.5%)	153	
Chest Pain	3 (1.8%)	0 (0.0%)	3	
Cough	42 (25.0%)	98 (46.2%)	140	
Dyspnea	32 (19.1%)	14 (6.6%)	46	
Duration of symptoms before presentation				.48
≤ 24 hours	47 (25.8%)	62 (29.0%)	109	
> 24 hours	135 (74.2%)	153 (71.0%)	287	
Lung sounds				
Wheezes	29 (15.9%)	44 (20.2%)	73	.27
Crackles/rales	40 (22.0%)	29 (13.3%)	69	.02
No wheezes or crackles/rales documented	107 (58.8%)	152 (69.7%)	259	.02
Clinical severity in the emergency department				
Altered mental status†	1 (0.6%)	1 (0.5%)	2	.90
Dehydration†	18 (9.9%)	11 (5.1%)	29	.06
Dyspnea† or tachypnea	50 (27.5%)	58 (26.6%)	108	.85
Fever (> 38 °C)	82 (45.1%)	105 (48.2%)	187	.53
High-flow nasal cannula	6 (3.3%)	1 (0.5%)	7	.03
Hypotension	1 (0.6%)	0 (0.0%)	1	.27
Hypoxia (SpO_2_ < 90%)	24 (13.2%)	7 (3.2%)	31	< .001
Tachycardia	93 (51.1%)	99 (45.4%)	192	.26
Sepsis†				0.89
No	178 (98.3%)	214 (98.2%)	392	
Yes	3 (1.7%)	4 (1.8%)	7	
Fluid resuscitation for hemodynamic instability				< .001
Not required	161 (88.5%)	216 (99.1%)	377	
Required	21 (11.5%)	2 (0.9%)	23	
Intubation				-
Not required	182 (100.0%)	218 (100.0%)	400	
Required	0 (0.0%)	0 (0.0%)	0	
Laboratory studies				< .001
Not obtained	101 (56.1%)	170 (78.0%)	271	
Obtained	79 (43.9%)	48 (22.0%)	127	
Chest radiograph findings^				
Unilateral	155 (85.2%)	132 (60.1%)	287	< .001
Bilateral	21 (11.5%)	81 (37.2%)	102	< .001
Focal infiltrate	120 (65.9%)	134 (61.5%)	254	.36
Atelectasis	41 (22.5%)	20 (9.2%)	61	< .001
Pleural effusion	7 (3.9%)	5 (2.3%)	13	.36
Multifocal	43 (23.6%)	78 (35.8%)	121	.01
Aspiration	3 (1.7%)	0 (0.0%)	3	.06
Other indications for admission				
Dehydration	10 (5.5%)	11 (5.1%)	21	.84
Inability to tolerate oral intake	7 (3.9%)	5 (2.3%)	12	.36
Hypoxia (SpO_2_ <90%)	27 (14.8%)	12 (5.5%)	39	< .01
Respiratory distress	20 (11.0%)	19 (8.7%)	39	.46
Failure of outpatient antibiotics&	13 (7.1%)	6 (2.8%)	19	.04
Clinical worsening#	15 (8.2%)	4 (1.8%)	19	< .01
Family preference	1 (0.6%)	0 (0.0%)	1	.27
Comorbid presentation%	10 (5.5%)	4 (1.8%)	14	.05
Emergency department observation unit				< .001
No	154 (84.6%)	206 (94.5%)	360	
Yes	28 (15.4%)	12 (5.5%)	40	

Features of clinical diagnostic and treatment course for pediatric patients presenting to the emergency department who are diagnosed with pneumonia. Table denotes data extracted from individual patient chart review; it includes factors that may contribute to pneumonia severity and reason for admission.

* Corresponds to *t*-test for continuous variables and chi-square test or Fisher exact test (two-tailed; noted if used) for categorical variables.

-Not applicable or sample is too small.

† Marked as positive if the clinician documented such in their note.

*CED*, community emergency department; *PED*, pediatric emergency department; *SpO*_2_, oxygen saturation of peripheral blood.

^ Multiple descriptors extracted from radiologist report.

& Patient presents for clinical worsening or symptoms that persist longer than the original outpatient antibiotic course.

# Failure to improve clinical condition despite antibiotic and supportive therapies in the ED or marked decline in vital signs or laboratory values.

% Patient has pneumonia in addition to another acute medical problem such as appendicitis, seizure, gastroenteritis.

*CED*, community emergency department; *PED*, pediatric emergency department; *SpO*_2_, oxygen saturation of peripheral blood.
