# Supplementary material for: Differences in Admission Rates of Children with Pneumonia Between Pediatric and Community Emergency Departments
Source: West J Emerg Med. 2025 Nov 26;26(6):1729–37. doi: 10.5811/westjem.47221 (PMC12698177; doi:10.5811/westjem.47221)
Supplement: Supplementary file 1 [file wjem-26-1729-s001.docx]

Appendix 1: ICD-10 Codes for Pneumonia

| J12………………………………………………….……………………………………………………… Viral pneumonia  J13…..…………………………………………………….………………… Pneumonia due to Streptococcus pneumoniae  J14……………………………………………………………………………… Pneumonia due to Hemophilis inflluenzae  J15……………………………………………………………………………………..… Bacterial pneumonia, unspecified  J16………………………………………………………………………...…. Pneumonia due to other infectious organisms  J17….…………………………………………………………………………. Pneumonia in diseases classified elsewhere  J18……………………………………………………………………………………..… Pneumonia, unspecified organism | | |  |
| --- | --- | --- | --- |
|  |  |  |  |
